# Ethnic differences in health related quality of life for patients with type 2 diabetes

**DOI:** 10.1186/1477-7525-12-83

**Published:** 2014-06-05

**Authors:** Tracey Jhita, Stavros Petrou, Anil Gumber, Ala Szczepura, Neil T Raymond, Srikanth Bellary

**Affiliations:** 1 Division of Health Sciences, Warwick Medical School, The University of Warwick, Coventry CV4 7AL, UK; 2 Centre for Health and Social Care Research, Faculty of Health and Wellbeing, Sheffield Hallam University, Sheffield, UK; 3 Aston Research Centre for Healthy Ageing (ARCHA), Aston University, Birmingham, UK

**Keywords:** Type 2 diabetes, Health related quality of life, EQ-5D, Ethnicity, South Asian

## Abstract

**Background:**

The objective of this study was to investigate the association between ethnicity and health related quality of life (HRQoL) in patients with type 2 diabetes.

**Methods:**

The EuroQol EQ-5D measure was administered to 1,978 patients with type 2 diabetes in the UK Asian Diabetes Study (UKADS): 1,486 of south Asian origin (Indian, Pakistani, Bangladeshi or other south Asian) and 492 of white European origin. Multivariate regression using ordinary least square (OLS), Tobit, fractional logit and Censored Least Absolutes Deviations estimators was used to estimate the impact of ethnicity on both visual analogue scale (VAS) and utility scores for the EuroQol EQ-5D.

**Results:**

Mean EQ-5D VAS and utility scores were lower among south Asians with diabetes compared to the white European population; the unadjusted effect on the mean EQ-5D VAS score was −7.82 (Standard error [SE] = 1.06, p < 0.01) and on the EQ-5D utility score was −0.06 (SE = 0.02, p < 0.01) (OLS estimator). After controlling for socio-demographic and clinical confounders, the adjusted effect on the EQ-5D VAS score was −9.35 (SE = 2.46, p < 0.01) and on the EQ-5D utility score was 0.06 (SE = 0.04), although the latter was not statistically significant.

**Conclusions:**

There was a large and statistically significant association between south Asian ethnicity and lower EQ-5D VAS scores. In contrast, there was no significant difference in EQ-5D utility scores between the south Asian and white European sub-groups. Further research is needed to explain the differences in effects on subjective EQ-5D VAS scores and population-weighted EQ-5D utility scores in this context.

## Background

Diabetes is a chronic metabolic disease estimated to have affected 285 million people globally in 2010, with the prevalence expected to increase to 438 million people by 2030 [1]. In the UK, there were approximately 2.8 million people with some form of diabetes in 2010 and, of those, 90% had *type 2 diabetes*[1]. *Type 2 diabetes* prevalence is reported to be three to four times higher in south Asian adults and the disease may occur a decade earlier than in the white European majority population in the UK [2,3]. *Type 2 diabetes* is associated with micro and macro vascular complications, including increased risk of cardiovascular disease, diabetic nephropathy, retinopathy, neuropathy and lower extremity amputations [4]. *Type 2 diabetes* and its associated co-morbidities may impact on many areas of an individual’s life, including physical function, social interaction and mental well-being; patients may consequently experience impaired health related quality of life (HRQoL).

There is evidence to suggest that the prevalence, disease progression and treatment outcomes for people with *type 2 diabetes* vary significantly between ethnic groups [5-9], but very little research has been undertaken to date to directly assess the extent to which ethnicity impacts on HRQoL in adults with diabetes. In addition, in the few studies that have explored this relationship, there is inconsistency in the tools used to measure HRQoL and a lack of consensus about the optimum method of assessment. This, combined with differences in the black and minority ethnic (BME) populations studied, have resulted in both negative [5-7,9-11] and positive [12-14] associations being reported between ethnicity and HRQoL. Although ethnicity may have an independent effect on HRQoL for people with *type 2 diabetes*, the size of any effect is unclear. More accurate quantification of such an effect should provide policy makers and service providers with evidence to develop initiatives that improve HRQoL for those ethnic minority populations that are most detrimentally affected. Furthermore, government agencies in many nations, such as the National Institute for Health and Care Excellence (NICE) in England and Wales, rely on cost-utility analyses, which seek to maximise Quality Adjusted Life Year (QALY) based measures of benefit that contain a HRQoL component. If there are ethnic differences in how a disease such as diabetes affects HRQoL, this should be taken into account when considering the introduction of new interventions on cost-effectiveness grounds.

The aim of the study was to investigate the association between ethnicity and HRQoL in patients with *type 2 diabetes* where HRQoL was measured using visual analogue scale (VAS) and utility scores for the EuroQol EQ-5D measure [15].

## Methods

This research used data collected by the UK Asian Diabetes Study (UKADS), which comprised of a community-based, cluster randomized controlled trial that evaluated the effectiveness of a culturally sensitive enhanced care package in UK general practice in improving cardiovascular risk factors in south Asian patients with *type 2 diabetes*, plus a parallel cohort study of cardiovascular risk factors in south Asian and white European patients with *type 2 diabetes*[3]. The main study, carried out in 2004–2007, recruited a total of 1486 south Asian and 492 white European patients from 25 general practices in Birmingham (16 practices) and Coventry (9 practices). All adults with *type 2 diabet*es were eligible for inclusion in the study; people with type 1 diabetes or impaired glucose tolerance were excluded. The research reported in this paper uses a cross-sectional study design, comparing HRQoL data for south Asian and white European patients in UKADS at the baseline assessment.

HRQoL was measured using the EuroQol EQ-5D, which is the generic, multi-attribute, preference based measure preferred by NICE for cost-effectiveness comparative purposes [16,17]. The EQ-5D consists of two principal measurement components. The first is a descriptive system, which defines health-related quality of life in terms of five dimensions: ‘mobility’, ‘self care’, ‘usual activities’, ‘pain/discomfort’ and ‘anxiety/depression’. Responses in each dimension are divided into three ordinal levels coded: (1) no problems; (2) some or moderate problems; and (3) severe or extreme problems. A total of 243 (3^5^) health states are generated by the EQ-5D descriptive system. For the purposes of this study, the York A1 (Dolan) tariff was applied to each set of responses to generate an EQ-5D utility score (preference weight) for each patient [16]. The York A1 tariff set was derived from a survey of the UK general population (*n* = 3337) which used the time trade-off valuation method to estimate utility scores for a subset of 45 EQ-5D health states, with the remainder of the EQ-5D health states subsequently valued through the estimation of a multivariate model [17]. Resulting utility scores range from scores −0.59 to 1.0, with 0 representing death and 1.0 representing full health; values below 0 indicate health states worse than death. The second measurement component of the EQ-5D consists of a 20 cm vertical visual analogue scale ranging from 100 (best imaginable health state) to 0 (worst imaginable health state), which provides an indication of the subject’s own assessment of their health status on the day of the survey.

Descriptive statistics were produced for baseline characteristics and analysed by ethnic meta-group (white European vs. south Asian) and by south Asian subgroup (Indian, Pakistani, Bangladeshi and other south Asian). Chi-squared and t-tests were employed to compare baseline variables between ethnic meta-groups and between ethnic subgroups. A descriptive analysis of levels of function within each EQ-5D dimension by ethnicity was complemented by further analysis of sub-optimal levels of function (defined as level 2 or 3) for each EQ-5D dimension. Fisher’s exact test for equality of proportions was used to compare sub-optimal levels of function within each EQ-5D dimension between ethnic meta-groups.

Four alternative methods of multivariate analysis were used to model the association between EQ-5D VAS or utility scores (dependent variables) and ethnicity: ordinary least squares (OLS), Tobit, fractional logit (Flogit) [18] and Censored Least Absolutes Deviations (CLAD) [19]. OLS regression is the most widely used of these estimators in the literature. It relies on the Gauss Markov assumptions about the data and variables used in the model, which need to be met in order to produce unbiased estimators [20]. Tobit regression was used to account for the non-trivial proportion of the study population with maximum EQ-5D VAS scores or EQ-5D utility scores [20,21]. The main assumptions of the Tobit model are that the error term has a normal distribution and that it has constant variance. If there is heteroskedasticity present, this may lead to inconsistent estimators being produced. Alternatively, when there are specification issues with the Tobit model, Powell’s Censored Least Absolutes Deviation (CLAD) estimator can be applied. The CLAD estimator is a generalisation of the least absolute deviations estimator [19]. The fractional logit model (Flogit) [18] provides a further alternative to the OLS and Tobit estimators. The Flogit model considers the dependent variable as a count, rather than a continuous variable, by using logit transformation and binomial regression. There is no single empirical test to evaluate the performance of these alternative estimators, so we developed an *a priori* plan based on comparable studies. Our aim was to investigate whether one estimator provided consistently more accurate estimates. Mean absolute errors (MAEs) and root mean squared errors (RMSEs) were used to examine differences between predicted and observed EQ-5D VAS and utility scores and to also check model performance [22]. All models were tested for heteroskedasticity using the White test [20]. Once a suitable model had been chosen, bi-variate analyses were conducted to estimate the unadjusted effects of ethnicity on EQ-5D VAS and utility scores. Partially adjusted and fully adjusted multi-variate models were then estimated. In the partially adjusted multivariate models, additional independent variables included age (continuous variable), gender (male, female), marital status (single, married, divorced, widowed) and the Carstairs deprivation index (categorical variable, see Table 1 for categories). The Carstairs index is an index of deprivation, based on four census indicators: low social class, lack of car ownership, overcrowding and male unemployment. In the original UKADS data, patient post-codes were collected, which were linked to the area specific Carstairs deprivation index, with higher scores representing elevated deprivation and vice versa [23]. In the fully adjusted models, further independent variables included body mass index (BMI) (18.5-24.9, <18.5, 25.0-29.9, 30.0-34.9, 35.0-39.9, >40.0), smoking status (non-smoker, current smoker, ex-smoker), alcohol use (yes, no), duration of diabetes in years (0–1, 2–5, 6–10, 11–15, >15), treatment regime (diet, tablets, insulin, insulin and tablets), family history of diabetes (yes, no), history of cardiovascular disease (CVD) (yes, no), albumin concentration (proteinuria and microalbuminuria were defined as urinary albumin to creatinine ratio (UACR) > =30.0 mg/mmol for both males and females and UACR 2.5 – <30.0 mg/mmol for males and 3.5- < 30.0 mg/mmol for females, respectively, with values below these thresholds indicating normal albuminuria) and GP practice.

**Table 1:** Characteristics of the study population by ethnicity

	**Indians**	**Pakistanis**	**Bangladeshis**	**Other South Asians**	**All South Asians**	**White Europeans**	**p-value†**
	**(n = 461)**	**(n = 876)**	**(n = 138)**	**(n = 11)**	**(n = 1486)**	**(n = 492)**	
**Age (years)**							
Mean [SD]	59.5 [11.4]	56.2 [11.9]	54.3 [11.5]	49.5 [11.5]	57.0 [11.9]	64.8 [11.6]	<0.0001
**Gender**							
Male; n (%)	267 (58.0)	438 (50.0)	65 (47.1)	6 (54.6)	776 (52.2)	287 (58.6)	0.015
Female; n (%)	193 (42.0)	438 (50.0)	73 (52.9)	5 (45.5)	709 (47.7)	203 (41.4)	
**Marital status**							
Single; n (%)	8 (1.8)	2 (0.2)	0 (0.0)	1 (9.1)	11 (0.7)	38 (8.0)	<0.0001
Married; n (%)	402 (90.1)	811 (93.0)	128 (92.8)	9 (81.8)	1350 (90.8)	358 (75.1)	
Widowed; n (%)	31 (7.0)	53 (6.1)	9 (6.5)	1 (9.1)	94 (6.3)	49 (10.0)	
Divorced; n (%)	5 (1.1)	6 (0.7)	1 (0.7)	0 (−)	12 (0.8)	32 (6.7)	
**Carstairs index score**							
Mean [SD]	2.9 [3.6]	8.2 [4.0]	8.2 [4.1]	9.0 [4.6]	6.6 [4.6]	1.7 [3.1]	<0.0001
−5 – 0.49, n (%)	133 (29.0)	51 (5.9)	8 (5.8)	0 (0.0)	192 (13.0)	202 (42.1)	
0.5 – 3.9, n (%)	159 (34.7)	74 (8.6)	14 (10.2)	2 (18.2)	249 (16.9)	160 (33.3)	
4.0 – 6.9, n (%)	97 (21.2)	184 (21.3)	32 (23.4)	2(18.2)	315(21.4)	83 (17.3)	
7.0 – 9.9, n (%)	62 (13.5)	257 (29.7)	39 (28.5)	1 (9.1)	359(24.4)	32 (6.7)	
10.0 – 17.0, n (%)	7 (1.5)	300 (34.6)	44 (32.1)	6(54.6)	357(24.3)	3 (0.6)	
**Smoking status**							
Non-smoker; n (%)	407 (88.3)	634 (72.4)	89 (64.5)	5 (45.5)	1135 (76.4)	231 (47.0)	<0.0001
Current smoker; n (%)	29 (6.3)	156 (17.8)	36 (26.1)	2 (18.2)	223 (15.0)	75 (15.2)	
Ex-smoker; n (%)	25 (5.4)	86 (9.8)	13 (9.4)	4 (36.4)	128 (8.6)	186 (37.8)	
**Alcohol use**							
No; n (%)	298 (64.6)	858 (97.9)	138 (100)	10 (90.9)	1304 (87.8)	231 (47.0)	<0.0001
Yes; n (%)	163 (35.4)	18 (2.1)	0 (−)	1 (9.1)	182 (12.2)	261 (53.0)	
**BMI score**							
Mean [SD]	28.3 [5.0]	28.9 [5.3]	26.5 [4.0]	29.6 [5.6]	28.5 [5.1]	30.9 [7.3]	<0.0001
< 18.5; n (%)	1 (0.2)	2 (0.2)	0 (−)	0 (−)	3 (0.2)	5 (1.0)	
18.5 – 24.9; n (%)	88 (19.1)	156 (17.8)	46 (33.3)	2 (18.2)	292 (19.7)	74 (15.0)	
25.0 – 29.9; n (%)	218 (47.3)	370 (42.2)	64 (46.4)	4 (36.4)	656 (44.2)	144 (29.3)	
30.0 – 34.9; n (%)	106 (23.0)	231 (26.4)	20 (14.5)	3 (27.3)	360 (24.2)	156 (31.7)	
35.0 – 39.9; n (%)	36 (7.8)	86 (9.8)	7 (5.1)	2 (18.2)	131 (8.8)	57 (11.6)	
>40.0; n (%)	12 (2.6)	31 (3.5)	1 (0.7)	0 (0.0)	44 (3.0)	56 (11.4)	
**Duration of diabetes (years)**							
Mean [SD]	7.6 [7.0]	7.7 [6.7]	8.5 [6.1]	3.9[4.6]	7.7 [6.7]	6.3 [7.0]	<0.0001
0 – 1years, n (%)	80 (17.4)	126 (14.4)	7 (5.1)	2 (18.2)	215 (14.5)	74 (15.0)	
2 – 5years, n (%)	154 (33.4)	304 (34.7)	51 (37.0)	8 (72.7)	517 (34.8)	226 (45.9)	
6 – 10years, n (%)	113 (24.5)	215 (24.5)	31 (22.5)	0 (−)	359 (24.2)	111 (22.6)	
11 – 15years, n (%)	50 (10.9)	116 (13.2)	29 (21.0)	0 (−)	195 (13.1)	41 (8.3)	
>15years, n (%)	64 (13.9)	115 (13.1)	20 (14.5)	1 (9.1)	200 (13.5)	40 (8.1)	
**Diabetes treatment**							
Diet only; n (%)	48 (10.4)	113 (12.9)	13 (9.4)	2 (18.2)	176 (11.8)	142 (28.9)	<0.0001
Tablets; n (%)	328 (71.2)	587 (67.0)	97 (70.3)	8 (72.7)	1038 (69.9)	285 (57.9)	
Insulin; n (%)	47 (10.2)	73 (8.3)	12 (8.7)	1 (9.1)	128 (8.6)	35 (7.1)	
Tablets and Insulin; n (%)	38 (8.2)	103 (11.8)	16 (11.6)	0 (−)	144 (9.7)	30 (6.1)	
**Family history of diabetes**							
No; n (%)	236 (51.2)	386 (44.1)	75 (54.3)	3 (27.3)	700 (47.1)	287 (58.3)	<0.0001
Yes; n (%)	225 (48.8)	490 (55.9)	63 (45.7)	8 (72.7)	786 (52.9)	205 (41.7)	
**History of cardiovascular disease**							
No; n (%)	371 (80.5)	724 (82.7)	114 (82.6)	9 (81.8)	1218 (82.0)	387 (78.7)	0.104
Yes; n (%)	90 (19.5)	152 (17.4)	24 (17.4)	2 (18.2)	268 (18.0)	105 (21.3)	
**Albumin concentration**							
Normal; n (%)	303 (72.8)	612 (73.6)	85 (64.4)	7 (77.8)	1007 (72.5)	343 (74.1)	<0.0001
Microalbuminuria; n (%)	84 (20.2)	162 (19.5)	33 (25.0)	2 (22.2)	281 (20.2)	109 (23.5)	
Proteinuria; n (%)	29 (7.0)	58 (7.0)	14 (10.6)	0 (−)	101 (7.3)	11 (2.4)	
**EQ-5D utility score**							
Mean [SD]	0.8 [0.3]	0.6 [0.4]	0.7 [0.3]	0.6 [0.4]	0.7 [0.3]	0.7 [0.3]	0.0005
**EQ-5D VAS score**							
Mean [SD]	63.7 [19.7]	63.2 [22.3]	64.0 [21.4]	69.2 [28.2]	63.5 [21.49]	71.3 [18.1]	<0.0001

Missing data: Age 3, gender 3, marital status 34, Carstairs Index 26, proteinuria 126, EQ-5D utility score 272, EQ-5D VAS score 306.

† Comparisons between all south Asians and white Europeans using Student t-tests for continuous variables and χ^2^ test for categorical variables.

Finally to allow for potential clustering effects and confounding, the SAS PROC MIXED procedure was separately used to fit hierarchical, combined fixed and random effects models. Random effects were fitted, within a subject term for general practice, allowing for different intercepts for all individual practices (random intercepts models).

The bulk of the analyses were conducted using SPSS 19.0, STATA 11 and SAS 9.3. Differences were considered statistically significant if p-values were less than 0.05.

## Results

Table 1 describes the characteristics of each ethnic group. The south Asian sample was younger than the white European group, with a mean age of 57 (standard deviation (SD) 11.9) vs. 64.8 (SD 11.6); this difference was statistically significant (p < 0.001). Duration of diabetes was significantly longer (p < 0.001) for the south Asian compared with the white European sample (Table 1). More south Asians were treated with insulin or a combination of insulin and tablets, compared with white Europeans (18.3% vs. 13.2%), while more white Europeans controlled their diabetes solely through diet (28.9% vs. 11.8%). These patterns were also observed in the study by Bellary *et al.*[24].

A family history of diabetes was recorded for 50% of patients, with the proportion significantly higher for south Asians compared to their white European counterparts. 18% of South Asians had some history of cardiovascular disease and approximately 28% had some indication of nephropathy (Table 1). However, the presence of chronic heart disease and microalbuminuria was higher in the white European sample, whilst proteinuria was more common amongst south Asians, in comparison to white Europeans.

Most study participants had BMI scores that were above normal (88.6%), with a high proportion of the white European sample classed as obese (54.7%). Using WHO cut-offs for Asian populations, 14% of south Asians were overweight (BMI of 23–24.9) and 80% were obese (BMI of ≥25) [25]. The majority of the sample was non-smokers; however, more white Europeans than south Asians were ex-smokers (37.8% vs. 8.6%). Current alcohol consumption was reported for 182 (12.2%) south Asians compared to 261 (53.0%) of the white Europeans. The mean Carstairs index was significantly higher (indicating residence in areas of higher deprivation) for south Asians at 6.6 (SD 4.6) compared to the white European sample, whose mean score was 1.7 (SD 3.1) (Table 1). Of the south Asian subgroups, Pakistani and Bangladeshi patients were more likely to reside in highly deprived areas.

Table 2 shows proportions of patients with sub-optimal levels of function within EQ-5D dimensions by ethnicity. For all dimensions except mobility a higher proportion of south Asians experienced sub-optimal function compared to their white European counterparts. Most notably, over 50% of the south Asian and white European meta-groups reported sub-optimal function for the pain/discomfort dimension, although the difference between ethnic meta-groups was not statistically significant. For the dimensions of anxiety/depression and usual activities, the proportion of south Asians reporting sub-optimal levels of function was significantly higher (p = 0.01 for usual activities, p = 0.02 for anxiety/depression) compared to their white European counterparts.

**Table 2:** **Number (%) of patients with sub-optimal levels of function**^**§ **^**within each EQ-5D dimension**

**Dimension**	**Indians**	**Pakistanis**	**Bangladeshis**	**Other South Asians**	**All South Asians**	**White Europeans**	**p-value**^**†**^
	**(n = 461)**	**(n = 876)**	**(n = 138)**	**(n = 11)**	**(n = 1486)**	**(n = 492)**	
Mobility; n (%)	78 (20.9)	373 (48.3)	45 (36.9)	4 (40.0)	500 (39.1)	198 (45.6)	0.017
Self care; n (%)	44 (11.7)	165 (21.4)	25 (20.5)	3 (30.0)	237 (18.5)	64 (14.8)	0.074
Usual activities; n (%)	80 (21.3)	322 (41.9)	42 (34.4)	4 (40.0)	448 (35.1)	123 (28.3)	0.010
Pain/Discomfort; n (%)	175 (46.9)	529 (68.7)	80 (65.6)	7 (70.0)	791 (62.0)	250 (57.6)	0.102
Anxiety/Depression; n (%)	86 (22.9)	290 (37.7)	35 (28.7)	4 (40.0)	415 (32.5)	115 (26.5)	0.020

Missing values: Mobility 266, Self care 265, usual activities 269, Pain/Discomfort 269, Anxiety/Depression 277.

§Sub-optimal levels of function defined as levels 2 or 3 for each EQ-5D dimension.

^†^Calculated using Fisher’s exact test for equality of proportions comparing south Asians and white European.

Model diagnostics comparing the different estimators for the fully adjusted multivariate models are summarised in online Additional file 1: Table S1 (EQ-5D VAS scores) and online Additional file 1: Table S2 (EQ-5D utility scores). The estimator with the smallest MAE and RMSE values shows the best fit for the data [22]. The CLAD estimator had the largest MAE and RMSE values. The results for the remaining estimators were broadly comparable across model type. The White test rejected the hypothesis of homoscedasticity in the error term (p < 0.01) for all estimators; robust standard errors (SEs) were used to correct for this. For ease of interpretation, we report the results for the OLS estimator in our main tables with comparative results across estimators found in online Additional file 1: Table S3 and S4.

Tables 3 and 4 respectively present results for the OLS unadjusted, partially adjusted and fully adjusted models for the EQ-5D VAS and EQ-5D utility scores. For EQ-5D VAS scores, the impact of being of south Asian descent was a decrement of 7.82 compared to the white European counterparts (SE 1.06; p < 0.01) (Table 3). For EQ-5D utility scores, south Asian descent was, on average, associated with a decrement in utility score of 0.06 (SE 0.02, p < 0.01) (Table 4). After controlling for socio-economic and socio-demographic variables, a significant inverse association remained between south Asian ethnicity and EQ-5D VAS scores [−7.30 (SE 1.36, p < 0.01)]. In contrast, a non-significant association was found between south Asian ethnicity and EQ-5D utility scores. The fully adjusted analyses strengthened the effect of south Asian ethnicity on EQ-5D VAS scores [−9.35 (SE 2.46, p <0.01)] (Table 3), whilst the effect on EQ-5D utility scores remained insignificant [0.06 (SE 0.04)] (Table 4). Results of the separate mixed effects models, which included random intercepts for individual general practices, were very similar to the final OLS estimates; for EQ-5D VAS scores −9.31, p = 0.0002 and for EQ-5D utility scores −0.02, p = 0.5751.

**Table 3:** Ordinary least squares estimator of marginal effects for EQ-5D VAS scores

	**Unadjusted β [Robust SE] (n = 1706)**	**Partially adjusted β [Robust SE] (n = 1654)**	**Fully adjusted β [Robust SE] (n = 1545)**
**Ethnicity (Referent = White Europeans)**			
All South Asians	-0.06 [0.02]**	0.02 [0.02]	0.06 [0.04]
**Age (years)**		-0.002 [0.001]*	-0.002 [0.001]**
**Gender (Referent = Male)**			
Female		-0.10 [0.02]**	-0.10 [0.02]**
**Marital status (Referent = Single)**			
Married		-0.08 [0.03]**	-0.07 [0.03]*
Widowed		-0.03 [0.04]	-0.02 [0.04]
Divorced		-0.14 [0.07]*	-0.18 [0.06]**
**Ethnicity (Referent = White Europeans)**			
**Carstairs index (Referent = 10.0-17.0)**			
-5 – 0.49		0.20 [0.03]**	0.05 [0.03]
0.5 – 3.9		0.16 [0.03]**	0.02 [0.03]
4.0 – 6.9		0.10 [0.03]**	0.01 [0.03]
7.0 – 9.9		0.08 [0.03]**	0.04 [0.03]
**Smoking status (Referent = Non Smoker)**			
Current smoker			-0.02 [0.02]
Ex-smoker			-0.05 [0.02]*
**Alcohol use (Referent = No)**			
Yes			0.003 [0.02]
**BMI score (Referent 18.5 – 24.9)**			
< 18.5			-0.19 [0.13]
25.0 – 29.9			-0.02 [0.02]
30.0 – 34.9			-0.08 [0.02]**
35.0 – 39.9			-0.14 [0.03]**
>40.0			-0.18 [0.04]**
**Duration of diabetes (Referent = 0-1 years)**			
2 – 5 years			-0.04 [0.02]
6 – 10 years			-0.04 [0.03]
11 – 15 years			-0.06 [0.03]
>15 years			-0.04 [0.03]
Tablets			-0.004 [0.02]
Insulin			-0.08 [0.04]*
Tablets and Insulin			-0.05 [0.03]
**Family history of diabetes (Referent = No)**			
Yes			0.01 [0.02]
**History of chronic heart disease (Referent = No)**			
Yes			-0.07 [0.02]**
**Albumin concentration (Referent = Normal)**			
Microalbuminuria			0.03 [0.02]
Proteinuria			-0.03 [0.04]
**Constant**	0.73 [0.01]**	0.79 [0.05]**	0.89 [0.08]**

*p-value <0.05; **p-value <0.01.

**Table 4:** Ordinary least squares estimator of marginal effects for EQ-5D utility scores

	**Unadjusted β [Robust SE] (n = 1706)**	**Partially adjusted β [Robust SE] (n = 1654)**	**Fully adjusted β [Robust SE] (n = 1545)**
**Ethnicity (Referent = White Europeans)**			
All South Asians	-0.06 [0.02]**	0.02 [0.02]	0.06 [0.04]
**Age (years)**		-0.002 [0.001]*	-0.002 [0.001]**
**Gender (Referent = Male)**			
Female		-0.10 [0.02]**	-0.10 [0.02]**
**Marital status (Referent = Single)**			
Married		-0.08 [0.03]**	-0.07 [0.03]*
Widowed		-0.03 [0.04]	-0.02 [0.04]
Divorced		-0.14 [0.07]*	-0.18 [0.06]**
**Carstairs index (Referent = 10.0-17.0)**			
-5 – 0.49		0.20 [0.03]**	0.05 [0.03]
0.5 – 3.9		0.16 [0.03]**	0.02 [0.03]
4.0 – 6.9		0.10 [0.03]**	0.01 [0.03]
7.0 – 9.9		0.08 [0.03]**	0.04 [0.03]
**Smoking status (Referent = Non Smoker)**			
Current smoker			-0.02 [0.02]
Ex-smoker			-0.05 [0.02]*
**Alcohol use (Referent = No)**			
Yes			0.003 [0.02]
**BMI score (Referent 18.5 – 24.9)**			
< 18.5			-0.19 [0.13]
25.0 – 29.9			-0.02 [0.02]
30.0 – 34.9			-0.08 [0.02]**
35.0 – 39.9			-0.14 [0.03]**
>40.0			-0.18 [0.04]**
**Duration of diabetes (Referent = 0-1 years)**			
2 – 5 years			-0.04 [0.02]
6 – 10 years			-0.04 [0.03]
11 – 15 years			-0.06 [0.03]
>15 years			-0.04 [0.03]
**Diabetes treatment (Referent = Diet only)**			
Tablets			-0.004 [0.02]
Insulin			-0.08 [0.04]*
Tablets and Insulin			-0.05 [0.03]
**Family history of diabetes (Referent = No)**			
Yes			0.01 [0.02]
**History of chronic heart disease (Referent = No)**			
Yes			-0.07 [0.02]**
**Albumin concentration (Referent = Normal)**			
Microalbuminuria			0.03 [0.02]
Proteinuria			-0.03 [0.04]
**Constant**	0.73 [0.01]**	0.79 [0.05]**	0.89 [0.08]**

*p-value <0.05; **p-value <0.01.

Unadjusted, partially adjusted and fully adjusted analyses were also conducted for south Asian subgroups and can be found in online Additional file 1: Table S5 and S6. In the fully adjusted model, all south Asian ethnicities were associated with significantly lower EQ-5D VAS scores compared to the white European ethnic origin (online Additional file 1: Table S5); the most significant decrement was observed for patients of Indian origin. In contrast, when the EQ-5D utility scores were analysed by ethnic sub-group, the fully adjusted model revealed no significant decrements in scores for each of the south Asian ethnicities compared to those of white European ethnic origin (online Additional file 1: Table S6).

## Discussion

In this study, the baseline disease profile of the south Asian patients mirrored that reported in the broader literature; younger people with a longer disease duration, increased severity of disease (as measured by type of treatment), and a higher incidence of family history of the disease, compared to the white European population. However, indications of CVD and albumin concentration were slightly lower in the south Asian group. This may be attributable to the population being younger and also to lifestyle factors, as south Asian patients did not smoke as much and consumed less alcohol than the white European sample. However, the percentage of current smokers in the sample (15%) was less than the current smoking population in England (21%) [26]. Similarly, the percentage of people who consumed alcohol in the entire sample (22%) was less than the comparative figure for England (63%) [27]. The south Asian population resided in areas with higher levels of deprivation than the white European sample. Mean EQ-5D VAS and utility scores were lower than for white European patients; with more of the south Asian population reporting sub-optimal levels of function across the self care, usual activities, and pain and anxiety/depression dimensions of the EQ-5 D. Mobility was the one dimension for which the White European sample reported higher sub-optimal levels of function. This could be related to the fact that the White European sample had a mean age of 64 years compared to a mean age of 57 years for the south Asians.

Our regression analyses indicate that south Asian ethnicity has an overall negative impact on EQ-5D VAS scores in patients with *type 2 diabetes*. On average, EQ-5D VAS scores were 9.35 lower for south Asians compared to white European patients in the fully adjusted OLS model. Comparison of the ethnic subgroups showed that Indians experienced the largest negative decrement in EQ-5D VAS scores compared to their white European counterparts in the adjusted analyses. Lower EQ-5D utility scores in south Asians were also observed, with south Asians experiencing an adjusted utility decrement of 0.06 compared to the white European sample. However, the effects on EQ-5D utility scores were only significant in the unadjusted analyses. It should be noted that a difference of 0.05 in mean multi-attribute utility scores is deemed clinically important for evaluative purposes [28]. The results of the regression analyses for both the EQ-5D VAS and utility scores remained robust to alternative model estimators.

The study revealed a consistent negative association between south Asian ethnicity and the EQ-5D VAS scores in patients with *type 2 diabetes*. In the broader literature, there are few studies that used the EQ-5D measure to assess HRQoL in ethnic minority populations with diabetes. Of the studies that did use this measure, Lubetkin *et al.*[14] reported results similar to the present study. The authors had used the EQ-5D measure to compare HRQoL between African-Americans, Hispanics and white Americans and found that after adjusting for known determinants of HRQoL, including chronic conditions such as diabetes; Hispanics had lower EQ-5D VAS scores and slightly higher EQ-5D utility scores compared to the white American sample. However, African Americans experienced both higher EQ-5D VAS and utility scores compared to the white American population. Although these results are similar in some respects to the ones reported here, they may not be generalisable due to the fact that different ethnic minority populations were studied. Differences may also be attributable to other methodological factors; for example, Lubetkin *et al.*[14] controlled for income, education and six chronic conditions in their study. In the current study, no data were available for income or education although lifestyle factors, co-morbidities and area deprivation were all included. In addition, cultural differences in rating tendencies have been observed in some studies. Lubetkin *et al.*[14] discussed the possibility that US Asians may be averse to valuing their health at the upper end of the VAS scale and may choose values towards the middle of the scale. This could partly account for any consistently negative association between ethnic minority status and EQ-5D VAS scores. In contrast, for EQ-5D utility scores, although individuals’ describe their own health-related quality of life, the values placed on those descriptions reflect general population preferences for health states. Moreover, a different valuation technique (the time trade-off approach) is used to derive those values. In the present study, there were patients who reported low EQ-5D VAS scores, such as a score of 20, but who had EQ-5D utility scores at the upper end of the utility scale, for example 0.8. Clearly, further research is required that disentangles the effects of the valuation technique itself and the underpinning source of values when assessing ethnicity-related effects on the HRQoL of individuals with type 2 diabetes.

One caveat to this study was the absence of information on education levels in the two samples. A lower level of education has been shown to be associated with impaired HRQoL [14]. Furthermore, as diabetes is a self-managed disease, higher levels of education could relate to better self-management and control of the disease, which in its turn should promote HRQoL. A study by Thoolen *et al.* examined the characteristics of patients with diabetes who participate in diabetes self-management programmes [29]. The authors found that education level was the most important factor differentiating participants and non-participants/dropouts, with participation linked to higher education levels. Since the south Asian sample in our study, on average, resided in areas with higher levels of deprivation compared to the White European sample, it is possible that their educational levels were also lower than in the white Europeans. By not adjusting for this difference between the two groups in the regressions, education effects may be reflected in the error terms of our models. A second caveat to the study revolves around the estimators selected to model the association between ethnicity and EQ-5D VAS or utility scores. Our selection of OLS, Tobit, Flogit and CLAD estimators was in keeping with the modelling approaches used more broadly by health economists to estimate EQ-5D values [16,30]. Furthermore, our statistical plan for model selection, which included the use of MAEs, RMSEs and tests for heteroskedasticity was in keeping with recommended diagnostic approaches [22]. Nevertheless, a number of methodological concerns surround the application of these estimators when modelling EQ-5D data. The OLS estimator does not capture the ceiling effect at 1.0 for utility values, whilst the Tobit and CLAD estimators may generate biased results when the true utility is conceptually bounded above at 1.0 [31,32]. The FLogit estimator has not previously been used on an extensive basis in the modelling utility data literature; a recent application did not generate promising results [33]. Alternative estimators for modelling health state utility values, such as adjusted limited dependent variable mixture models, are currently under development [34]; their application to UKADS data remains a topic for future enquiry.

## Conclusion

The study suggests that south Asian ethnicity has a negative effect on both the EQ-5D VAS and utility scores compared to white Europeans. However no statistically significant difference was observed for utility scores when socio-economic, demographic and clinical variables were included in the models. Moreover, as observed in the broader literature, the nature and magnitude of the HRQoL effects vary depending on the ethnic minority under consideration [12,14]. Further research is needed to explain the differences in ethnicity-related effects on subjective EQ-5D VAS scores and population-weighted EQ-5D utility scores in patients with *type 2 diabetes*.

## Competing interests

The authors declare that they have no competing interests.

## Authors’ contributions

TJ was responsible for the day to day conduct of the study, including additional data collection over and above the original data collection within UKADS, analysis and writing. SP and AS had the original idea for the study; together with AG they also provided oversight for the study. AG, NR and SB contributed to the research design, interpretation of results and writing. All authors read and approved the final manuscript.

## Supplementary Material

Additional file 1: Table S1 Estimated predicted scores compared to observed EQ-5D VAS scores (fully adjusted model for all south Asians). **Table S2.** Estimated predicted scores compared to observe EQ-5D utility scores (fully adjusted model for all south Asians). **Table S3.** Marginal effects for EQ-5D VAS scores for all south Asians^†^ by estimator and model. **Table S4.** Marginal effects for EQ-5D utility scores for all south Asians^†^ by estimator and model. **Table S5.** OLS marginal effects of sub-group south Asian ethnicity on EQ-5D VAS scores^†±^. **Table S6.** OLS marginal effects of sub-group south Asian ethnicity on EQ-5D utility scores^†±^. Click here for file
