# Supplementary material for: Ethnic differences in health related quality of life for patients with type 2 diabetes
Source: Health Qual Life Outcomes. 2014 Jun 5;12:83. doi: 10.1186/1477-7525-12-83 (PMC4060842; doi:10.1186/1477-7525-12-83)
Supplement: Additional file 1: Table S1 — Estimated predicted scores compared to observed EQ-5D VAS scores (fully adjusted model for all south Asians). Table S2. Estimated predicted scores compared to observe EQ-5D utility scores (fully adjusted model for all south Asians). Table S3. Marginal effects for EQ-5D VAS scores for all south Asians† by estimator and model. Table S4. Marginal effects for EQ-5D utility scores for all south Asians† by estimator and model. Table S5. OLS marginal effects of sub-group south Asian ethnicity on EQ-5D VAS scores†±. Table S6. OLS marginal effects of sub-group south Asian ethnicity on EQ-5D utility scores†±. [file 1477-7525-12-83-S1.docx]

**Additional file 1**

**Table S1: Estimated predicted scores compared to observed EQ-5D VAS scores (fully adjusted model for all south Asians)**

| **Model** | **Mean** | **Min** | **Max** | **MAE^†^** | **RMSE^§^** |
| --- | --- | --- | --- | --- | --- |
| Observed VAS score | 65.48 | 6 | 100 |  |  |
| **Predicted score** |  |  |  |  |  |
| OLS | 65.80 | 37.62 | 104.01 | 15.27 | 19.30 |
| Tobit | 65.41 | 36.68 | 95.90 | 14.98 | 18.53 |
| Flogit | 65.42 | 40.59 | 93.19 | 15.08 | 18.69 |
| CLAD | 68.57 | 32.46 | 98.57 | 17.37 | 21.72 |

†Mean absolute error; §Root mean squared error

**Table S2: Estimated predicted scores compared to observed EQ-5D utility scores (fully adjusted model for all south Asians)**

| **Model** | **Mean** | **Min** | **Max** | **MAE^†^** | **RMSE^§^** |
| --- | --- | --- | --- | --- | --- |
| Observed utility score | 0.68 | -0.43 | 1 |  |  |
| **Predicted score** |  |  |  |  |  |
| OLS | 0.69 | 0.24 | 1.14 | 0.226 | 0.30 |
| Tobit | 0.68 | 0.10 | 0.98 | 0.223 | 0.29 |
| Flogit | 0.68 | 0.13 | 0.97 | 0.223 | 0.29 |
| CLAD | 0.79 | 0.35 | 1.05 | 0.230 | 0.33 |

†Mean absolute error; §Root mean squared error

**Table S3: Marginal effects for EQ-5D VAS scores for all south Asians^†^ by estimator and model**

|  | **OLS**  **β [Robust SE]** | **Tobit**  **β [Robust SE]** | **Flogit**  **β [Robust SE]** | **CLAD**  **β [Robust SE]** |
| --- | --- | --- | --- | --- |
| **Unadjusted model** | -7.82 [1.06] ** | -7.28** [1.08] | -8.55** [1.20] | -15.00** [0.509] |
| Constant [Robust SE] | 71.29 [0.87] ** | 71.94 [0.94] |  | 75.00 [0.100] |
| **Partially adjusted model§** | -7.30 [1.36] ** | -6.63** [1.35] | -7.99** [1.51] | -10.40**[1.87] |
| Constant [Robust SE] | 85.68 [3.96] ** | 80.76 [4.42] |  | 82.40 [4.54] |
| **Fully adjusted model#** | -9.35 [2.46] ** | -10.42** [3.03] | -10.58**[2.79] | -13.45** [8.41] |
| Constant [Robust SE] | 81.21 [6.09] ** | 77.55 [7.05] |  | 79.16 [13.90] |

†Base category = White Europeans

§ Covariates controlled for included age, gender, marital status and Carstairs Index.

# Covariates controlled for included age, gender, marital status, Carstairs Index, smoking status, alcohol use, BMI score, duration of diabetes, diabetes treatment, family history of diabetes, history of chronic heart disease, albumin concentration and GP practice.

* p-value <0.05; ** p-value <0.01

**Table S4: Marginal effects for EQ-5D utility scores for all south Asians^†^ by estimator and model**

|  | **OLS**  **β [Robust SE]** | **Tobit**  **β [Robust SE]** | **Flogit**  **β [Robust SE]** | **CLAD**  **β [Robust SE]** |
| --- | --- | --- | --- | --- |
| **Unadjusted model** | -0.06 [0.02] ** | -0.04* [0.016] | -0.04** [0.012] | -0.04* [0.024] |
| Constant [Robust SE] | 0.73 [0.01] ** | 0.80 [0.019] |  | 0.80 [0.020] |
| **Partially adjusted model§** | 0.02 [0.02] | 0.05* [0.021] | 0.02 [0.015] | 0.05 [0.018] |
| Constant [Robust SE] | 0.79 [0.05] | 0.73 [0.074] |  | 0.79 [0.050] |
| **Fully adjusted model#** | 0.06 [0.04] | 0.04 [0.062] | 0.08* [0.036] | 0.01* [0.022] |
| Constant [Robust SE] | 0.89 [0.08] ** | 0.95 [0.001] |  | 0.87 [0.094] |

†Base category = White Europeans

§ Covariates controlled for included age, gender, marital status and Carstairs Index.

# Covariates controlled for included age, gender, marital status, Carstairs Index, smoking status, alcohol use, BMI score, duration of diabetes, diabetes treatment, family history of diabetes, history of chronic heart disease, albumin concentration and GP practice.

* p-value <0.05; ** p-value <0.01

**Table S5: OLS marginal effects of sub-group south Asian ethnicity on EQ-5D VAS scores^†±^**

|  | **Unadjusted**  **β [Robust SE]** | **Partially adjusted§**  **β [Robust SE]** | **Fully adjusted#**  **β [Robust SE]** |
| --- | --- | --- | --- |
| **Indian** | -7.62 [1.34] ** | -7.60 [1.46] ** | -9.96 [2.47] ** |
| **Pakistani** | -8.08 [1.20] ** | -7.25 [1.71] ** | -9.14 [3.07] ** |
| **Bangladeshi** | -7.29 [2.13] ** | -6.66 [2.44] ** | -8.26 [3.30] * |
| **Constant** | 71.29 [0.87]** | 85.06 [4.08] ** | 80.16 [6.30] ** |

†Base category = White Europeans

±Other south Asian group removed from subgroup analysis due to size of group (n=11)

§ Covariates controlled for included age, gender, marital status and Carstairs Index.

# Covariates controlled for included age, gender, marital status, Carstairs Index, smoking status, alcohol use, BMI score, duration of diabetes, diabetes treatment, family history of diabetes, history of chronic heart disease, albumin concentration and GP practice.

* p-value <0.05; ** p-value <0.01

**Table S6: OLS marginal effects of sub-group south Asian ethnicity on EQ-5D utility scores^†±^**

|  | **Unadjusted**  **β [Robust SE]** | **Partially adjusted§**  **β [Robust SE]** | **Fully adjusted#**  **β [Robust SE]** |
| --- | --- | --- | --- |
| **Indian** | 0.08 [0.02] ** | 0.09 [0.02] ** | 0.07 [0.04] |
| **Pakistani** | -0.13 [0.02] ** | -0.10 [0.03] ** | 0.04 [0.05] |
| **Bangladeshi** | -0.004 [0.04] | 0.004 [0.04] | 0.08 [0.05] |
| **Constant** | 0.73 [0.01]** | 0.88 [0.05]** | 0.91 [0.09]** |

†Base category = White Europeans

±Other south Asian group removed from subgroup analysis due to size of group (n=11)

§ Covariates controlled for included age, gender, marital status and Carstairs Index.

# Covariates controlled for included age, gender, marital status, Carstairs Index, smoking status, alcohol use, BMI score, duration of diabetes, diabetes treatment, family history of diabetes, history of chronic heart disease, albumin concentration and GP practice.

* p-value <0.05; ** p-value <0.01
